# Occurrence and management of postoperative pneumocephalus using the semi-sitting position in vestibular schwannoma surgery

**DOI:** 10.1007/s00701-020-04504-5

**Published:** 2020-07-25

**Authors:** Kathrin Machetanz, Felix Leuze, Kristin Mounts, Leonidas Trakolis, Isabel Gugel, Florian Grimm, Marcos Tatagiba, Georgios Naros

**Affiliations:** Department of Neurosurgery, Eberhardt Karls University, Hoppe-Seyler-Straße 3, 72076 Tuebingen, Germany

**Keywords:** Tension pneumocephalus, Intracranial air, Vestibular schwannoma, Semi-sitting position, Supine position

## Abstract

**Background:**

The semi-sitting position in neurosurgical procedures is still under debate due to possible complications such as venous air embolism (VAE) or postoperative pneumocephalus (PP). Studies reporting a high frequency of the latter raise the question about the clinical relevance (i.e., the incidence of tension pneumocephalus) and the efficacy of a treatment by an air replacement procedure.

**Methods:**

This retrospective study enrolled 540 patients harboring vestibular schwannomas who underwent posterior fossa surgery in a supine (*n* = 111) or semi-sitting (*n* = 429) position. The extent of the PP was evaluated by voxel-based volumetry (VBV) and related to clinical predictive factors (i.e., age, gender, position, duration of surgery, and tumor size).

**Results:**

PP with a mean volume of 32 ± 33 ml (range: 0–179.1 ml) was detected in 517/540 (96%) patients. The semi-sitting position was associated with a significantly higher PP volume than the supine position (40.3 ± 33.0 ml [0–179.1] and 0.8 ± 1.4 [0–10.2], *p* < 0.001). Tension pneumocephalus was observed in only 14/429 (3.3%) of the semi-sitting cases, while no tension pneumocephalus occurred in the supine position. Positive predictors for PP were higher age, male gender, and longer surgery duration, while large (T4) tumor size was established as a negative predictor. Air exchange via a twist-drill was only necessary in 14 cases with an intracranial air volume > 60 ml. Air replacement procedures did not add any complications or prolong the ICU stay.

**Conclusion:**

Although pneumocephalus is frequently observed following posterior fossa surgery in semi-sitting position, relevant clinical symptoms (i.e., a tension pneumocephalus) occur in only very few cases. These cases are well-treated by an air evacuation procedure. This study indicates that the risk of postoperative pneumocephalus is not a contraindication for semi-sitting positioning.

## Introduction

The use of semi-sitting position in neurosurgery is still under debate [21, 24]. While the semi-sitting position facilitates surgery by providing a clean surgical field and enabling a bimanual preparation [9, 16, 17, 24, 28], there is a general reservation due to potential complications such as venous air embolism (VAE), intraoperative hypotension, or postoperative pneumocephalus (PP) [1, 13, 21]. Several neurosurgical and anesthesiological studies have shown a rather small risk for cardiovascular relevant VAE [7, 23] when specific precautions are taken. However, little is known about postoperative pneumocephalus. The pathophysiology of postoperative pneumocephalus is hypothesized to be based on the intraoperative loss of cerebrospinal fluid (CSF) and the subsequent intracranial entry of air, which corresponds to the “inverted soda-pop bottle” mechanism [4, 27, 29].

As the loss of CSF is a gravity-dependent phenomenon, postoperative pneumocephalus is more frequently found following neurosurgical procedures under semi-sitting position in comparison with the supine position [30]. Some studies report an occurrence rate of postoperative pneumocephalus up to 100% [6, 30]; however, only in a few cases this is accompanied by relevant clinical symptoms (e.g., loss of consciousness, seizures, or focal neurologic deficits), which define a postoperative tension pneumocephalus. The incidence of symptomatic postoperative pneumocephalus in semi-sitting position may vary between 3% [27] and 0.11% [10]. These studies highlight the fact that a suboccipital craniotomy with dura opening, in contrast to other surgeries in semi-sitting position (e.g., cervical interventions or deep brain stimulation), predicts the occurrence of postoperative pneumocephalus. Thus, although the sitting position may indeed favor the development of postoperative pneumocephalus, other factors may also contribute to its pathogenesis. We hypothesize that the opening of the basal cisterns in suboccipital craniotomy with CSF loss may be a strong source of postoperative pneumocephalus. In order to systematically address to this issue, two groups of patients who underwent retrosigmoid craniotomy under semi-sitting position (group 1) or supine position (group 2) were compared in regard to the appearance of postoperative pneumocephalus. All patients harbored the same type of tumor (vestibular schwannomas) and underwent the same surgical technique (retrosigmoid transmeatal approach).

The aim of this retrospective study is to describe the occurrence, predictors, and management of postoperative pneumocephalus after VS removal via a retrosigmoid approach in semi-sitting position in comparison with supine position. We hypothesize that there is a positive correlation between PP and tumor size determining the opening of arachnoidal plains during surgery. For the first time, the extent of postoperative pneumocephalus is evaluated by voxel-based morphometry and related to patients’ characteristics and surgical technique (e.g., position, surgery duration, tumor size, patient’s age).

## Methods

### Patients

This retrospective study enrolled a consecutive series of 540 patients (47.5 ± 14.1 years, 273 female) undergoing a neurosurgical removal of a VS in the Neurosurgical Department of the University of Tuebingen between January 2011 and March 2017. Preoperatively, all patients received a semi-structured interview of VS-associated symptoms by an experienced neurosurgeon, a hearing evaluation by an ENT specialist (pure tone audiogram and speech discrimination), and measurement of auditory evoked potentials as well as a magnetic resonance (MR) imaging of the brain. All surgeries were performed by the senior surgeon (MT) and another 3 surgeons trained by the first one. Consequently, all operations were carried out using the same technique. Patients’ characteristics are summarized in Table 1. The study was approved by the local ethics committee of the Eberhardt Karls University Tuebingen and performed in accordance with the Declaration of Helsinki.

**Table 1:** Data overview

Age	47.5 ± 14.1
Gender (f:m)	273:267
Tumor size	
T1 T2 T3 T4	22/540 (4%) 113/540 (21%) 218/540 (40%) 187/540 (35%)
Type of positioning	
Supine Semi-sitting	111/540 (21%) 429/540 (79%)
Surgical procedure time	
Overall Supine Semi-sitting	305 ± 77 min 283 ± 62 min 310 ± 80 min
Postoperative pneumocephalus (PP)	
Overall	517/540 (96%) 32 ± 33 ml (0–179.1)
Supine	89/111 (80.2%) 0.8 ± 1.4 ml (0–10.2)
Semi-sitting	428/429 (99.8%) 40.3 ± 33 ml (0–179.1)
Tension pneumocephalus	
Overall Supine Semi-sitting	14/540 (2.6%) 0/111 (0%) 14/429 (3.3%)

### Tumor size classification

In all patients, a preoperative magnetic resonance image (MRI) of the brain with gadolinium contrast was utilized and tumor extent was graded according to the Hannover classification [17]. VS were classified into 4 classes: T1 (purely intrameatal), T2 (intra- and extrameatal), T3a and T3b (filling the cerebellopontine cistern or touching the brain stem), T4a and T4b (compressing or shifting the brain stem).

### Intraoperative positioning

Selection of the position was dependent on tumor size. The majority of patients with T1 and T2 tumors are operated in supine position whereas T3 and T4 tumors are operated in semi-sitting position.

### Supine position

In the supine position, the head is fixed by means of a Mayfield skull clamp with the single pin at the forehead hairline laterally on the tumor side, and the double pin dorsally at the center line (i.e., using the inion as a landmark) or slightly contralateral. Subsequently, the head is rotated approximately 80° to the contralateral side and marginally tilted backwards. The shoulders end at the top of the operating table and, contrary to frequent textbook documentation, are not padded on the operating side.

### Semi-sitting position

**Fig. 1:**
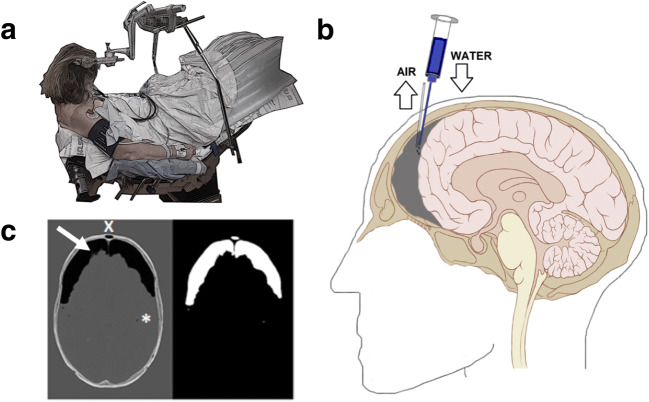
**a** Semi-sitting position in VS surgery. **b** Evacuation of postoperative tension pneumocephalus by twist-drill trepanation. **c** Postoperative pneumocephalus is recognized as intracranial air-isodense voxels (Hounsfield value − 1000) occurring mainly in the prefrontal region (Mount Fuji sign, arrow) and occasionally in the cisterns (air bubble sign, asterisk). A custom-made algorithm for voxel-based volumetry detected intracranial air automatically. However, air volumes within paranasal sinus (x) had to be removed manually by point-and-click

The semi-sitting position which has already been described elsewhere [17] was performed according to our protocol outlined by Tatagiba et al. [28]: The head is fixed by the Mayfield Clamp with the single pin on the ipsilateral side of lesion, ventrally to the tip of the ear. The body is positioned, such that the hip is flexed to a maximum of 90°, with the shoulders ending at the top of the operating table. To reduce the risk of air embolism, the legs are raised above the level of the heart (Fig. 1a). Afterwards, the head is fixed in an anteposition, 30–40° rotated to the ipsilateral side of the lesion and inclined with slight tilt towards the sternum. Finally, access to the jugular veins should be ensured to enable a jugular compression during surgery and to prevent venous outflow obstruction.

### Surgical procedure

The principles of the surgical procedure were unchanged throughout all patients and correspond to the procedure as described elsewhere in detail [28]. After planning the incision, patients’ hairs were shaved locally followed by skin asepsis and sterile draping. Cutis, subcutis, superficial, and deep neck muscles are opened until the bone is visualized. Subsequently, emissary veins are coagulated and closed by bone wax to prevent air embolism. The area of the asterion is identified and the mastoid tip is exposed. Then, the craniotomy or craniectomy is performed followed by a partial mastoidectomy. The borders of the bony removal are defined laterally by the sigmoid sinus, superiorly by the transversal sinus, and inferiorly by the horizontal part of the posterior cranial fossa. The dura is incised in a semilunar fashion parallel to the sigmoid sinus. The inferior part of the cerebellum is gently elevated and the cerebellomedullary cistern (CMC) is exposed. The CMC is sharply opened to drain cerebrospinal fluid (CSF). The posterior aspect of the petrous bone is exposed and certain amount of its dura is removed above the Tuebingen line [2] to expose the posterior wall of the internal auditory canal (IAC). Latter is drilled away and the intracanalicular tumor content is resected under identification of cranial nerves VII and VIII. Tumor extending into the CPA is debulked with ultrasonic aspirator, then cranial nerves VII and VIII are dissected stepwise from the tumor surface using a bimanual preparation technique. Finally, hemostasis is done, dura is closed, the bone flap or a cranioplasty is reinserted, and wound is closed.

Patients are usually extubated and monitored for 1 night on an intensive care unit (ICU) and receive a CT scan within 24 h after surgery. In cases with a delayed wake-up reaction (i.e., > 1 h after surgery), the CT scan is performed within a few hours after surgery. In cases with extensive pneumocephalus suggesting a tension pneumocephalus, air replacement is performed on ICU.

### Air replacement procedure (twist-drill evacuation)

In cases of a tension pneumocephalus, after locally shaving the hair at the forehead hairline (usually on the right side) and skin asepsis, a stab incision and 5-mm drill hole is made. Afterwards, the dura is carefully punctured with two cannulas, where a frontal trajectory must be chosen to prevent injury to the brain. Subsequently, the air is replaced with Ringer solution by connecting one of the cannulas with a syringe filled with water. This method enables a pressureless and complete air replacement (Fig. 1b). After air replacement, the cannulas are removed and the wound is closed.

### Voxel-based volumetry

A postoperative axial CT scan (resolution 3 mm, multi-slice CT scanner, Siemens Medical GmbH) was performed for each patient within 24 h after surgery to exclude any operative complication. Postoperative pneumocephalus is recognized as intracranial air-isodense voxel (Hounsfield value − 1000) mainly occurring in the prefrontal region (Mount Fuji sign, arrow) [18] and occasionally in the cisterns (air bubble sign, asterisk) [12] (Fig. 1c). VBV of postoperative supratentorial intracranial air volume was assessed by custom-made Matlab scripts (Version R2019a, MathWorks, Natick, MA, USA). The most challenging situation for automated detection was the discrimination between intracranial air and the frontal sinus. To overcome this, bone-weighted CT images were imported into the Matlab software, and all voxels with extracranial air were automatically removed based on their Hounsfield value (− 1000). Subsequently, manual removal of all slices below the frontal base was performed in order to avoid bias from the ethmoidal cells. Finally, the frontal sinus was denoted and removed manually (Fig. 1c).

### Statistical analysis

All analyses and statistical tests were performed using MATLAB (MathWorks, Inc., Natick, MA, USA) and SPSS (IBM SPSS Statistics for Windows, Version 26.0. Armonk, NY: IBM Corp.). Group differences between the supine and semi-sitting positions were evaluated by a non-parametric Kruskal-Wallis test. In order to ascertain the effects of positioning (POS; 0: supine and 1: semi-sitting), gender (GENDER; 0: female and 1: male), age (AGE), tumor size (T1–T4), and surgery time (TIME) on the volume of postoperative pneumocephalus, a multivariate linear regression was performed (using a STEPWISE approach). Data are shown as the mean ± standard deviation (SD). *p* values < 0.05 were considered significant. Finally, operative complications detected in the postoperative CT scan were evaluated.

## Results

### Characteristics of the patient cohort and the surgical procedure

This retrospective study included 540 patients (47.5 ± 14.1 years, 273 female) undergoing a neurosurgical removal of a VS. The proportions of tumor extent according to the Hannover classification are illustrated in Table 1. One hundred eleven out of 540 (21%) patients were operated in the supine position while 429/540 (79%) were operated in semi-sitting position. The rate of small tumors (i.e., T1 and T2) was significantly higher in the supine than in the semi-sitting position (*X*^2^ = 241.95, *p* < 0.001; Kruskal-Wallis). Ninety-one out of 135 (67%) of the small tumors were operated in supine position whereas 385/405 (95%) of the large tumors were operated in semi-sitting position. The mean surgical procedure time (SPT) was 305 ± 77 min, whereby approx. 90 min (for performing and closing the retrosigmoid transmeatal approach) must be deducted from the SPT to calculate the intradural surgery time. However, there was a significant difference in SPT depending on the position of the patient (supine: 283 ± 62 min; semi-sitting: 310 ± 80 min; *X*^2^ = 12.53, *p* < 0.001; Kruskal-Wallis). Cohort characteristics are summarized in Table 1.

### Volume of postoperative pneumocephalus

**Fig. 2:**
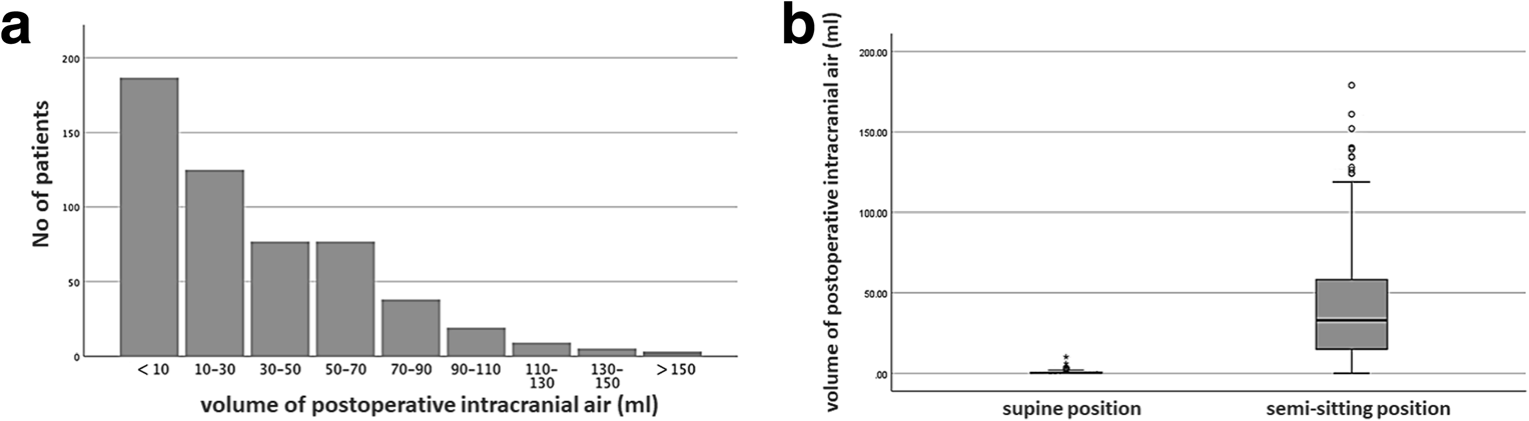
**a** Volume distribution of the postoperative pneumocephalus. **b** Patients operated in the semi-sitting position had a significant higher volume of postoperative pneumocephalus than patients that were operated in the supine position

Supratentorial intracranial air (Vol > 0 ml) was detected in 517/540 (96%) patients. The extent of pneumocephalus was assessed in the postoperative CT scan by VBV. The mean postoperative intracranial air was 32 ± 33 ml (range: 0–179.1 ml). The volume distribution of the postoperative pneumocephalus is shown in Fig. 2a. Five hundred twelve out of 517 (99%) patients exhibited a subdural air collection, while in contrast, intraventricular air was only seen in 5/517 patients (1%). Patients operated in the semi-sitting position had a significant higher volume of postoperative pneumocephalus than patients who were operated in the supine position (40.3 ± 33.0 ml [0–170.1] and 0.8 ± 1.4 ml [0–10.2], *p* < 0.001; Kruskal-Wallis; Fig. 2b).

### Predictors of postoperative pneumocephalus

In order to ascertain the effects of positioning (POS; 0: supine and 1: semi-sitting), gender (GENDER; 0: female and 1: male), age (AGE), tumor size (T1-T4), and surgery time (TIME) on the volume of postoperative pneumocephalus, a multivariate linear regression was performed (using a STEPWISE approach). After 5 iterations, a significant regression was established (*r*^2^ = 0.286, *F* = 42.55, *p* < 0.001). Of the predictor variables, POS, AGE, T4 size, and TIME were statistically significant (Table 2). As expected, the semi-sitting position was the strongest positive predictor (*B* = 41.26, 95% CI [34.87–47.6], *β* = 0.496, *p* < 0.001) of postoperative pneumocephalus, followed by the patient’s age (*B* = 0.43, 95% CI [0.26–0.61], *β* = 0.182, *p* < 0.001) and the surgery time (*B* = 0.06, 95% CI [0.02–0.09], *β* = 0.134, *p* = 0.001), as well as male gender (*B* = 6.8, 95% CI [1.96–11.68], *β* = 0.102, *p* = 0.006). Notably, T4 tumors were a negative predictor of postoperative pneumocephalus (*B* = − 9.79, 95% CI [− 15.66 to − 3.93], *β* = − 0.139, *p* < 0.001).

**Table 2:** Multivariate linear regression

	*B*	SE *B*	95% CI		*β*	*p* value
*Constant*	− 86.835	9.540	− 105.575	− 68.094		0.000
Positioning	41.255	3.251	34.870	47.641	0.496	0.000
Age	0.431	0.089	0.257	0.606	0.182	0.000
Gender	6.819	2.474	1.959	11.680	0.102	0.006
T4 size	− 9.794	2.986	− 15.659	− 3.928	− 0.139	0.001
Surgery time	0.058	0.018	0.023	0.093	0.134	0.001

### Occurrence of postoperative tension pneumocephalus necessitating intervention

Although the postoperative pneumocephalus was very common, most of the patients had no neurological deficits and did not demand a neurosurgical intervention. A tension pneumocephalus which needed neurosurgical intervention was observed in 14/540 (2.6%) cases with all cases occurring in a semi-sitting position (14/429, 3.3%). Characteristic of these cases is a postoperative reduction of consciousness without other neurological symptoms (e.g., anisocoria, limb paresis, cranial nerve paresis), challenging extubation within the first 60 min after surgery. Hence, the postoperative CT scan is performed excluding other complications (e.g., bleedings) and the indication for air replacement is made. In these patients, air was replaced via the aforementioned air replacement procedure. There were no complications related to the air replacement procedure. All patients stayed one single day on ICU; thus, there was no difference in regard to the length of ICU stay compared with patients without air replacement (0.97 ± 0.8 days; *p* = 0.799; Kruskal-Wallis).

**Fig. 3:**
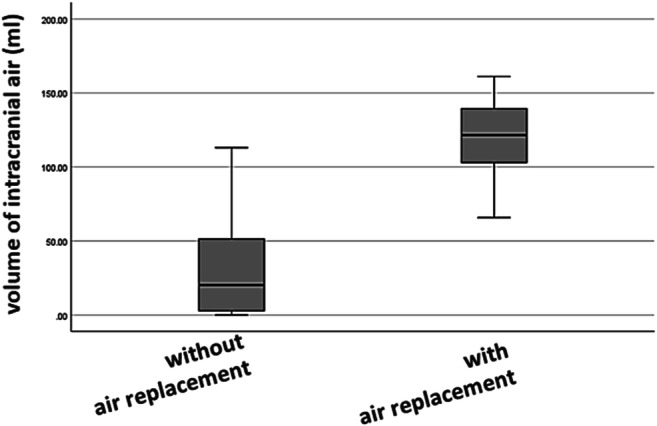
Relation between postoperative tension pneumocephalus and the volume of intracranial air

All patients presenting a postoperative tension pneumocephalus were operated in semi-sitting position and had an intracranial air of > 60 ml. However, there were also 5 patients (see outliers in Fig. 3) with a pneumocephalus of > 60 ml which did not receive air replacement. In 2 of these patients, extubation was delayed due to other complications (e.g., bleeding), while 3 of the remaining patients were extubated and recovered without air replacement, albeit with a prolonged stay in the ICU.

## Discussion

There still exists controversy in regard to the ideal positioning of patients during VS resection. While the semi-sitting position facilitates surgery, particularly in large tumors [16, 17, 24, 28], it creates conditions permitting complications such as VAE, intraoperative hypotension, or a postoperative pneumocephalus [1, 13, 16, 21, 28]. Within this context, the aim of our study was to evaluate the occurrence, clinical predictors, and management of a postoperative pneumocephalus in VS surgery, particularly using the semi-sitting positioning. For the first time, the extent of postoperative pneumocephalus was exactly measured by voxel-based volumetry in a large cohort.

Any amount of intracranial, mainly subdural, air collection was detected in 96% of the patients. The extent of postoperative pneumocephalus was significantly higher in semi-sitting position in comparison with supine position. All cases with postoperative tension pneumocephalus were observed following semi-sitting position procedures. While only a few studies in the literature have explored this paradigm, our findings are in line with the available data. Postoperative pneumocephalus in semi-sitting position is known to be very common and ranges in literature from 42.1% (40/95) [25] to 100% (30/30 and 32/32, respectively) of the cases [6, 30]. However, the incidence of symptomatic tension pneumocephalus has been shown to be infrequent, with reports varying between 3% (8/275) [27] and 0.11% (2/1792) [10]. In the present study, a postoperative tension pneumocephalus demanding air evacuation occurred in 2.6% of the cases (3.3% of the semi-sitting cases) with air volumes > 60 ml.

So far it remains unclear what turns asymptomatic to symptomatic pneumocephalus. It is hypothesized that the amount of air is decisive or at least indicative for the development of symptoms. However, there are only a few studies performing a quantitative analysis of the postoperative pneumocephalus [15, 19, 22]. Sloan et al. [25] describe an incidence of approximately 40% of postoperative pneumocephalus in 106 patients after posterior fossa surgery in semi-sitting position with a volume varying between 6 and 280 ml. However, although being symptomatic, none of these patients required treatment. In a similar manner to our results, Monajati et al. [19] reported in a small case study that an air volume of > 65 ml predicts a symptomatic pneumocephalus, while an air volume of < 20 ml was associated with an asymptomatic postoperative course. Thus, a volume of > 60 ml may represent a viable threshold for a postoperative tension pneumocephalus requiring neurosurgical intervention. In contrast, a lower air volume (48.5 ml) is observed in symptomatic cases when the postoperative pneumocephalus is confined to the intraventricular space [22].

While the exact pathophysiology of the postoperative pneumocephalus after semi-sitting surgery remains unresolved, it is usually explained by an increased drainage of CSF and the “inverted soda-bottle” mechanism described by Lunsford et al. [4]. In line, the risk of a postoperative pneumocephalus is not increased in deep brain stimulation surgeries despite being performed in semi-sitting surgery [10]. However, it remains unclear why suboccipital craniotomies have a significant higher risk of postoperative pneumocephalus in comparison with cervical spine surgeries in semi-sitting surgery [10, 27]. We hypothesize that the opening of the basal cisterns and the fourth ventricle at the level of the foramen of Luschka and Magendi after suboccipital craniotomy might facilitate CSF drainage from the inner and outer CSF spaces and correspondingly increase the risk of postoperative pneumocephalus. At the same time, opening the arachnoidal barriers might pave the way for the spread of the air. In line, while opening the fourth ventricle has been shown to be related to ventricular pneumocephalus, surgical interventions in the cerebellopontine angle predispose for subdural pneumocephalus [22]. This is in agreement with our results, in that most patients in the present study have shown subdural air collection, while intraventricular air was seen only in three patients.

Within this school of thought, a positive correlation between VS tumor size and subdural air collection is expected. However, our findings do not support this hypothesis. Firstly, correlation analysis between VS size and postoperative pneumocephalus is confounded by the fact that smaller tumors are usually operated in supine position. Secondly, the present study suggests T4 tumor size (i.e., compression of the brainstem by the vestibular schwannoma) as a negative predictor for a pneumocephalus. However, it has been shown that large (T4) tumors can occlude the ipsilateral foramen of Luschka and thereby impede further CSF loss from the fourth ventricle during VS surgery [14]. This could contribute to the reduced amount of subdural pneumocephalus in cases with a T4 tumor relative to cases with a T3 tumor during VS surgery in semi-sitting position.

Furthermore, the present study demonstrates that patient’s age, male gender, and the operation time are positive predictors for the occurrence of increased postoperative intracranial air. In line with this finding, surgeries with a intradural surgery duration longer than 4.5 h and performed on patients in a higher age have been shown to correlate with postoperative pneumocephalus [22, 25] Interestingly, male patients have been shown to hold a higher risk for postoperative pneumocephalus [22]. It is thought that the higher tendency for parenchymal atrophy in male subjects [3] as well as the higher CSF volumes in men may be contributing factors which allow for a higher amount of air to accumulate [22].

It is evident that subdural air collection after posterior fossa surgery is very common. However, the occurrence of a postoperative tension pneumocephalus which requires surgical air replacement is less likely in CPA surgery than in lesions affecting the fourth ventricle [5, 22]. Furthermore, the rare cases of postoperative tension pneumocephalus which do occur can be treated by a twist-drill air replacement, a procedure that holds a low complication rate and does not increase the ICU stay. The decision for air replacement should be based on clinical appearance (e.g., reduced level of consciousness, seizures, or focal neurological deficits). Asymptomatic or mild pneumocephalus could be treated conservatively with oxygen inhalation [8, 11, 20]. In this context, the present study does not analyze the time course of spontaneous air resorption and its influencing factors. Sachkova et al. [22] reported about 39.1% and Sloan et al. [25] about 19% remaining intracranial air on the third postoperative day. However, the significance of individual studies addressing the issue of air absorption is limited due to a small number of patients, populations that do not focus on patients with VS, or studies that focus on absorption behavior due to different ventilation modes [8, 11, 22, 26]. Consequently, further studies are necessary to obtain a greater significance in this respect.

## Conclusion

With a meticulous anesthesia and neurosurgical management, a pneumocephalus in VS resection requiring therapy is uncommon and does not contraindicate the use of semi-sitting position. The occurrence of pneumocephalus is attributed to the amount of CSF drainage from the inner and outer CSF spaces after opening the basal cisterns and the foramen of Luschka. Long surgery duration, elderly age, male gender, and smaller tumor size are positive predictors of the subdural air volume. However, it remains to be elucidated which major factors contribute to the occurrence of a symptomatic tension pneumocephalus.

## Abbreviations

CMC: Cerebellomedullary cistern
CSF: Cerebrospinal fluid
ICU: Intensive care unit
PP: Postoperative pneumocephalus
PTP: Postoperative tension pneumocephalus
SPT: Surgical procedure time
VAE: Venous air embolism
VBV: Voxel-based volumetry
VS: Vestibular schwannoma
